# Crystal structure and DFT computational studies of (*E*)-2,4-di-*tert*-butyl-6-{[3-(tri­fluoro­meth­yl)benz­yl]imino­meth­yl}phenol

**DOI:** 10.1107/S205698902000537X

**Published:** 2020-04-24

**Authors:** Nihal Kan Kaynar, Hasan Tanak, Mustafa Macit, Namık Özdemir

**Affiliations:** a Department of Physics, Faculty of Arts & Science, Amasya University, TR-05100, Amasya, Turkey; b Department of Chemistry, Faculty of Arts & Science, Ondokuz Mayıs University, TR-55139 Samsun, Turkey; c Faculty of Education, Department of Mathematics and, Science Education, Division of Physics Education, Ondokuz Mayıs University, TR-55139 Samsun, Turkey

**Keywords:** Schiff base, rotational disorder, enol–imine, *tert*-but­yl, DFT, crystal structure

## Abstract

The title compound, C_23_H_28_F_3_NO, is an *ortho*-hy­droxy Schiff base compound, which adopts the enol–imine tautomeric form in the solid state. The mol­ecular structure is not planar and the dihedral angle between the planes of the aromatic rings is 85.52 (10)°. The tri­fluoro­methyl group shows rotational disorder over two sites, with occupancies of 0.798 (6) and 0.202 (6). An intra­molecular O—H⋯N hydrogen bonding generates an *S*(6) ring motif. The crystal structure is consolidated by C—H⋯π inter­actions. The mol­ecular structure was optimized *via* density functional theory (DFT) methods with the B3LYP functional and LanL2DZ basis set. The theoretical structure is in good agreement with the experimental data. The frontier orbitals and mol­ecular electrostatic potential map were also examined by DFT computations.

## Abstract 2

The title Schiff base compound was synthesized and its crystal structure characterized by X-ray diffraction. The mol­ecular structure, frontier orbitals and mol­ecular electrostatic potential map were also investigated by DFT methods.

## Chemical context   

Schiff base ligands have played an important role in the development of coordination chemistry, specifically in relation to magnetism, enzymatic reactions (Moutet & Ourari, 1997 ▸) and mol­ecular architectures (Kaynar *et al.*, 2018 ▸). Schiff bases and their metal complexes have been used in anti­bacterial, anti­cancer, anti­fungal, anti­tubercular and hypothermic reagents (Marchant *et al.*, 1981 ▸; Turwatker & Mahta, 2007 ▸). Generally, *ortho*-hy­droxy Schiff base compounds display two tautomeric, enol–imine (OH) and keto–amine (NH), forms. Depending on the tautomers, two types of intra­molecular hydrogen bonds are observed in *ortho*-hy­droxy Schiff bases, namely, O—H⋯N in enol–imine and N—H⋯O in keto–amine tautomers (Tanak *et al.*, 2009 ▸, 2010 ▸). In this study, we report the synthesis, crystal structure and density functional theory (DFT) calculations of the title Schiff base compound.
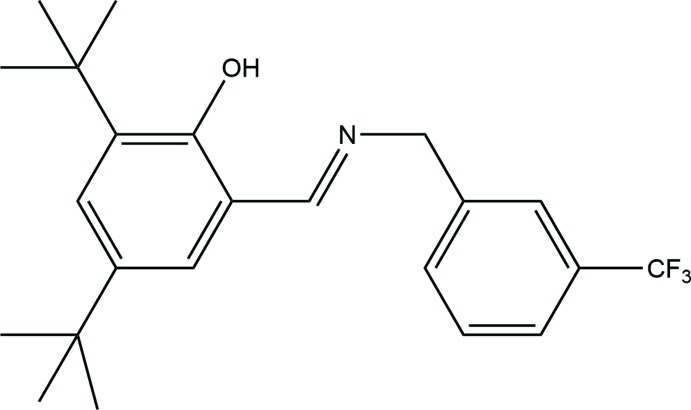


## Structural commentary   

**Figure 1:**
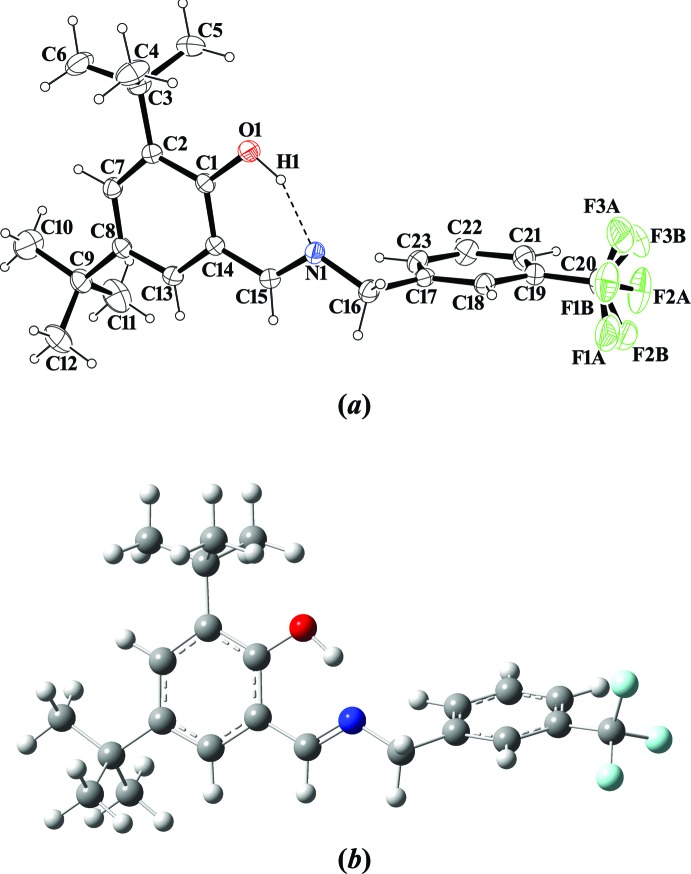
(*a*) The mol­ecular structure of the title compound, showing the atom-numbering scheme and 20% probability displacement ellipsoids and (*b*) the optimized mol­ecular structure of the title compound generated at the DFT/B3LYP/LanL2DZ level.

The mol­ecular structure of the title compound is shown in Fig. 1 ▸(*a*). The crystal structure is monoclinic and has the space-group type *P*2_1_/c. The CF_3_ group exhibits rotational disorder [Fig. 1 ▸(*a*)]. The site-occupancy factors are 0.798 (6) and 0.202 (6) for F1*A*/F2*A*/F3*A* and F1*B*/F2*B*/F3*B*, respectively. The DFT computations of the title compound were performed with the *Gaussian 09W* program package (Frisch *et al.*, 2009 ▸) using the B3LYP functional and the LanL2DZ basis set. The optimized mol­ecular structure is illustrated in Fig. 1 ▸(*b*). Some selected theoretical bond lengths, bond angles and torsion angles are given in Table 1 ▸ along with the experimental values. The mol­ecular structure of the title compound is not planar: the dihedral angle between the 2,4-di-*tert*-butyl­phenol and the tri­fluoro­methyl rings is 85.52 (10)°. This dihedral angle was calculated to be 65.73° for the B3LYP computationally derived structure. The imino group is nearly coplanar with the 2,4-di-*tert*-butyl­phenol ring, as indicated by the C1—C14—C15—N1 torsion angle [−3.9 (3)° for X-ray and −0.14° for B3LYP]. There is an intra­molecular O1—H1⋯N1 hydrogen bond present (Fig. 1 ▸ and Table 2 ▸), generating an *S*(6) ring motif. The C1—O1 bond length [1.353 (2) Å for X-ray and 1.376 Å for B3LYP] indicates single-bond character. The imine C15=N1 bond length [1.273 (2) Å for X-ray and 1.308 Å for B3LYP] indicates double-bond character. In the title compound, the bond lengths and bond angles are within normal ranges and they are comparable with those in related Schiff base structures (Li *et al.*, 2007 ▸; Sun *et al.*, 2007 ▸; Çelik *et al.*, 2009 ▸; Şahin *et al.*, 2009 ▸; Kansiz *et al.*, 2018 ▸). The C1—O1 and C15=N1 bond lengths confirm the enol–imine form of the title compound (Tanak, 2011 ▸; Kaynar *et al.*, 2018 ▸).

## Supra­molecular features   

**Figure 2:**
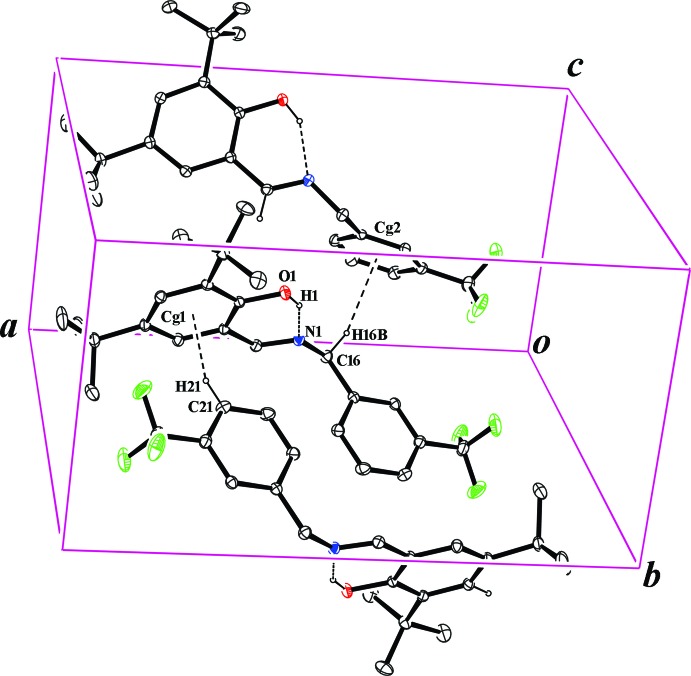
A partial packing plot of the title crystal. Dashed lines indicate the O—H⋯N intra­molecular hydrogen bonding and C—H⋯π inter­actions.

**Figure 3:**
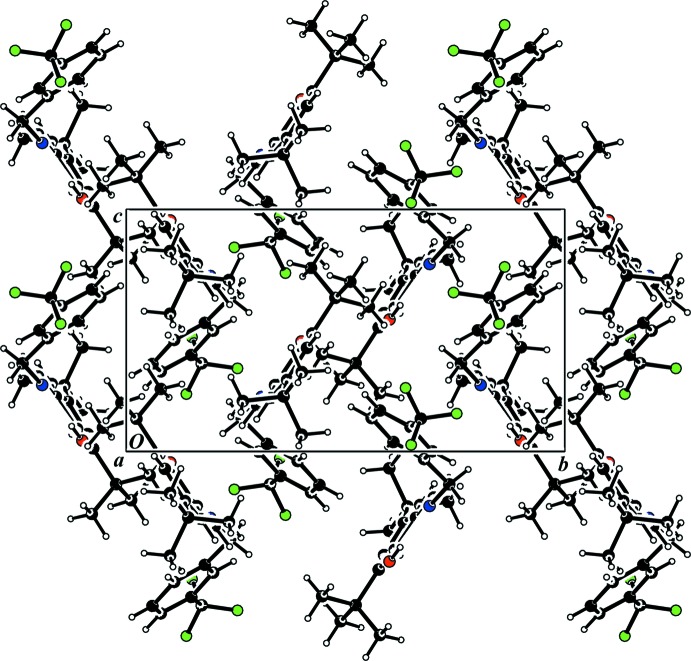
The crystal packing of the title compound, viewed along the *a* axis.

The crystal structure of the title compound is consolidated by C—H⋯π inter­actions (Fig. 2 ▸), details of which are summarized in Table 2 ▸. A packing diagram is shown in Fig. 3 ▸. The only other inter­actions are van der Waals contacts.

## Mol­ecular electrostatic potential (MEP)   

**Figure 4:**
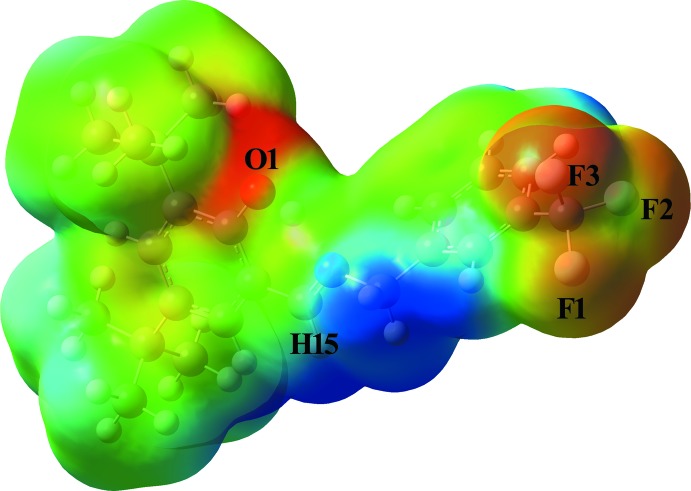
The mol­ecular electrostatic potential map of the title compound.

The mol­ecular electrostatic potential (MEP) is a very useful descriptor for classifying and understanding regions that are susceptible to electrophilic *versus* nucleophilic attack. In order to analyse reactive regions for electrophilic and nucleophilic reactions for the investigated Schiff base mol­ecule, the MEP surface was computed using the B3LYP/LanL2DZ basis set for the optimized geometry. In the MEP surface, the negative potential regions (red areas) are associated with electrophilic reactivity, while the positive potential regions (blue areas) are related to nucleophilic reactivity. The MEP surface of the compound is shown in Fig. 4 ▸. The negative MEP regions are mainly over the O1, F1, F2, and F3 atoms and have values of −0.049 a.u., −0.031 a.u., −0.032 a.u. and −0.035 a.u., respectively. The largest maximum positive MEP region is localized on atom H15, and has a value of +0.048 a.u. According to these results, the preferred sites for electrophilic attack are around the oxygen and fluorine atoms, while the preferred region for nucleophilic attack is the imine group C—H atom, H15.

## Frontier mol­ecular orbitals   

**Figure 5:**
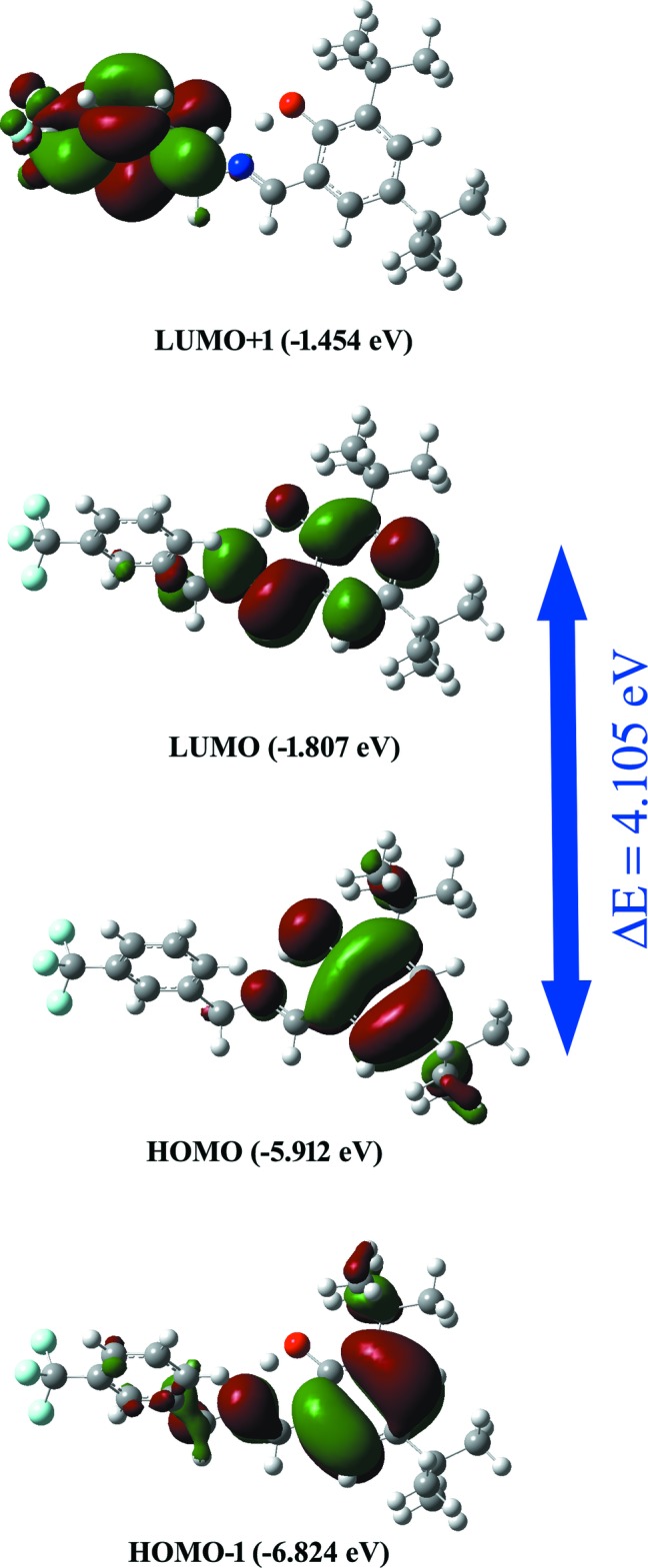
The frontier mol­ecular orbitals.

The highest occupied mol­ecular orbitals (HOMOs) and lowest unoccupied mol­ecular orbitals (LUMOs) are known as frontier mol­ecular orbitals. The electronic, optical and chemical reactivity properties of compounds are predicted by their frontier mol­ecular orbitals (Tanak, 2019 ▸). The frontier mol­ecular orbitals of the title compound were obtained using the DFT/B3LYP method with the LanL2DZ basis set. The energy levels and distributions of the frontier mol­ecular orbitals are shown in Fig. 5 ▸. The HOMO–LUMO gap is used to analyse the chemical reactivity and stability of a mol­ecule. If the mol­ecule has a large HOMO–LUMO gap, the mol­ecule is more stable and less chemically reactive. The term ‘hard mol­ecule’ is used to describe such cases. The electron affinity (*A* = -*E*_HOMO_), the ionization potential (*I* = -*E*_LUMO_), HOMO–LUMO energy gap (Δ*E*), the chemical hardness (η) and softness (*S*) of the title compound were predicted based on the *E*_HOMO_ and *E*_LUMO_ energies (Tanak, 2019 ▸). For the title compound, *I* = 5.912 eV, *A*= 1.807 eV, Δ*E* = 4.105 eV, η = 2.052 eV and *S* = 0.243 eV. As a result of the large Δ*E* and η values, the title compound can be classified as a hard mol­ecule.

## Synthesis and crystallization   

*(E)*-2,4-Di-*tert*-butyl-6-((3-(tri­fluoro­meth­yl)benzyl­imino)meth­yl)phenol was prepared by refluxing a mixture of a solution containing 3,5-di-*tert*-butyl-2-hy­droxy­benzaldehyde (46.8 mg, 0.2 mmol) in ethanol (30 ml) and a solution containing 3-(tri­fluoro­meth­yl)benzyl­amine (35.03 mg, 0.2 mmol) in ethanol (20 ml). The reaction mixture was stirred for 4 h under reflux. The title compound was obtained by slow evaporation of an ethanol solution (m.p. 401–403 K; yield 78%)

## Refinement   

Crystal data, data collection and structure refinement details are summarized in Table 3 ▸. C-bound H atoms were positioned geometrically and refined using a riding model, with C—H = 0.93–0.97 Å and *U*_iso_(H) = 1.2–1.5*U*_eq_(C). The position of the H1 atom was obtained from a difference map of the electron density in the unit cell and was refined freely.

## Supplementary Material

Crystal structure: contains datablock(s) I, namiko43. DOI: 10.1107/S205698902000537X/pk2624sup1.cif

Structure factors: contains datablock(s) I. DOI: 10.1107/S205698902000537X/pk2624Isup2.hkl

Supporting information file. DOI: 10.1107/S205698902000537X/pk2624Isup3.cml

CCDC reference: 1997654

Additional supporting information:  crystallographic information; 3D view; checkCIF report

## Figures and Tables

**Table 1:** Some selected bond lengths, bond angles and torsion angles (Å, °) in the experimentally determined and computed mol­ecular structures

Parameters	X-ray	DFT
O1—C1	1.353 (2)	1.376
N1—C15	1.273 (2)	1.308
N1—C16	1.457 (3)	1.467
C14—C15	1.456 (3)	1.457
		
C15—N1—C16	118.68 (18)	120.83
O1—C1—C2	119.87 (16)	120.63
O1—C1—C14	119.60 (17)	119.16
N1—C15—C14	122.99 (18)	121.96
N1—C16—C17	113.09 (16)	112.67
		
O1—C1—C14—C15	2.2 (3)	0.28
C16—N1—C15—C14	178.67 (18)	178.34
C13—C14—C15—N1	175.7 (2)	179.87
C15—N1—C16—C17	107.5 (2)	130.42

**Table 2:** Hydrogen-bond geometry (Å, °) *Cg*1 and *Cg*2 are the centroids of the C1/C2/C7/C8/C13/C14 and C17–C23 rings, respectively.

*D*—H⋯*A*	*D*—H	H⋯*A*	*D*⋯*A*	*D*—H⋯*A*
O1—H1⋯N1	0.92 (3)	1.73 (3)	2.587 (2)	154 (3)
C16—H16*B*⋯*Cg*2^i^	0.97	2.77	3.705 (3)	162
C21—H21⋯*Cg*1^ii^	0.93	2.85	3.631 (3)	143

Symmetry codes: (i) 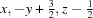; (ii) 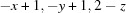.

**Table 3:** Experimental details

Crystal data	
Chemical formula	C_23_H_28_F_3_NO
*M*_r_	391.46
Crystal system, space group	Monoclinic, *P*2_1_/*c*
Temperature (K)	296
*a*, *b*, *c* (Å)	15.6783 (10), 15.7880 (14), 8.7054 (5)
β (°)	91.217 (5)
*V* (Å^3^)	2154.4 (3)
*Z*	4
Radiation type	Mo *K*α
μ (mm^−1^)	0.09
Crystal size (mm)	0.72 × 0.56 × 0.09

Data collection	
Diffractometer	STOE IPDS 2
Absorption correction	Integration (*X-RED32*; Stoe & Cie, 2002 ▸)
*T*_min_, *T*_max_	0.938, 0.992
No. of measured, independent and observed [*I* > 2σ(*I*)] reflections	24168, 4981, 2875
*R*_int_	0.062
(sin θ/λ)_max_ (Å^−1^)	0.652

Refinement	
*R*[*F* ^2^ > 2σ(*F* ^2^)], *wR*(*F* ^2^), *S*	0.059, 0.149, 1.01
No. of reflections	4981
No. of parameters	291
No. of restraints	67
H-atom treatment	H atoms treated by a mixture of independent and constrained refinement
Δρ_max_, Δρ_min_ (e Å^−3^)	0.17, −0.14

Computer programs: *X-AREA* and *X-RED32* (Stoe & Cie, 2002 ▸), *SHELXS97* and *SHELXL97* (Sheldrick, 2008 ▸) and *ORTEP-3 for Windows* and *WinGX* (Farrugia, 2012 ▸).

## supplementary crystallographic information

### Crystal data

**Table:**

C_23_H_28_F_3_NO	*F*(000) = 832
*M**_r_* = 391.46	*D*_x_ = 1.207 Mg m^−^^3^
Monoclinic, *P*2_1_/*c*	Mo *K*α radiation, λ = 0.71073 Å
*a* = 15.6783 (10) Å	Cell parameters from 19720 reflections
*b* = 15.7880 (14) Å	θ = 1.8–28.0°
*c* = 8.7054 (5) Å	µ = 0.09 mm^−^^1^
β = 91.217 (5)°	*T* = 296 K
*V* = 2154.4 (3) Å^3^	Plate, orange
*Z* = 4	0.72 × 0.56 × 0.09 mm

### Data collection

**Table:**

STOE IPDS 2 diffractometer	4981 independent reflections
Radiation source: fine-focus sealed tube	2875 reflections with *I* > 2σ(*I*)
Graphite monochromator	*R*_int_ = 0.062
Detector resolution: 6.67 pixels mm^-1^	θ_max_ = 27.6°, θ_min_ = 2.6°
rotation method scans	*h* = −20→20
Absorption correction: integration (X-RED32; Stoe & Cie, 2002)	*k* = −20→20
*T*_min_ = 0.938, *T*_max_ = 0.992	*l* = −10→11
24168 measured reflections	

### Refinement

**Table:**

Refinement on *F*^2^	Primary atom site location: structure-invariant direct methods
Least-squares matrix: full	Secondary atom site location: difference Fourier map
*R*[*F*^2^ > 2σ(*F*^2^)] = 0.059	Hydrogen site location: inferred from neighbouring sites
*wR*(*F*^2^) = 0.149	H atoms treated by a mixture of independent and constrained refinement
*S* = 1.01	*w* = 1/[σ^2^(*F*_o_^2^) + (0.072*P*)^2^] where *P* = (*F*_o_^2^ + 2*F*_c_^2^)/3
4981 reflections	(Δ/σ)_max_ < 0.001
291 parameters	Δρ_max_ = 0.17 e Å^−^^3^
67 restraints	Δρ_min_ = −0.14 e Å^−^^3^

### Special details

**Table:**

Experimental. 248 frames, detector distance = 80 mm
Geometry. All esds (except the esd in the dihedral angle between two l.s. planes) are estimated using the full covariance matrix. The cell esds are taken into account individually in the estimation of esds in distances, angles and torsion angles; correlations between esds in cell parameters are only used when they are defined by crystal symmetry. An approximate (isotropic) treatment of cell esds is used for estimating esds involving l.s. planes.
Refinement. Refinement of F^2^ against ALL reflections. The weighted R-factor wR and goodness of fit S are based on F^2^, conventional R-factors R are based on F, with F set to zero for negative F^2^. The threshold expression of F^2^ > 2sigma(F^2^) is used only for calculating R-factors(gt) etc. and is not relevant to the choice of reflections for refinement. R-factors based on F^2^ are statistically about twice as large as those based on F, and R- factors based on ALL data will be even larger.

### Fractional atomic coordinates and isotropic or equivalent isotropic displacement parameters (Å^2^)

**Table:**

	*x*	*y*	*z*	*U*_iso_*/*U*_eq_	Occ. (<1)
F1A	0.82817 (19)	0.75361 (18)	1.1564 (8)	0.1412 (18)	0.798 (6)
F2A	0.8487 (2)	0.6371 (4)	1.2628 (5)	0.1492 (18)	0.798 (6)
F3A	0.86847 (16)	0.6497 (4)	1.0276 (5)	0.1431 (18)	0.798 (6)
F1B	0.8509 (6)	0.7243 (11)	1.0355 (15)	0.108 (4)	0.202 (6)
F2B	0.8298 (5)	0.7109 (13)	1.2651 (14)	0.112 (4)	0.202 (6)
F3B	0.8690 (5)	0.6084 (6)	1.140 (2)	0.119 (4)	0.202 (6)
O1	0.42489 (9)	0.60037 (10)	0.54544 (18)	0.0583 (4)	
N1	0.47793 (10)	0.69181 (11)	0.77476 (19)	0.0517 (4)	
C1	0.34413 (11)	0.61230 (12)	0.5935 (2)	0.0442 (4)	
C2	0.27563 (12)	0.57558 (12)	0.5121 (2)	0.0474 (5)	
C3	0.28920 (14)	0.52313 (15)	0.3652 (2)	0.0604 (6)	
C4	0.3291 (2)	0.57867 (19)	0.2417 (3)	0.0900 (9)	
H4A	0.3843	0.5977	0.2770	0.135*	
H4B	0.2931	0.6268	0.2216	0.135*	
H4C	0.3350	0.5464	0.1490	0.135*	
C5	0.34662 (19)	0.44671 (17)	0.4018 (3)	0.0866 (8)	
H5A	0.4010	0.4662	0.4405	0.130*	
H5B	0.3546	0.4142	0.3101	0.130*	
H5C	0.3202	0.4120	0.4779	0.130*	
C6	0.20508 (18)	0.4890 (2)	0.2985 (3)	0.0923 (9)	
H6A	0.2160	0.4565	0.2078	0.138*	
H6B	0.1680	0.5354	0.2728	0.138*	
H6C	0.1784	0.4535	0.3732	0.138*	
C7	0.19492 (12)	0.58784 (13)	0.5719 (2)	0.0520 (5)	
H7	0.1487	0.5639	0.5192	0.062*	
C8	0.17838 (12)	0.63327 (13)	0.7047 (2)	0.0539 (5)	
C9	0.08888 (13)	0.64131 (16)	0.7727 (3)	0.0680 (6)	
C10	0.02115 (17)	0.5997 (3)	0.6718 (5)	0.1314 (15)	
H10A	−0.0338	0.6080	0.7159	0.197*	
H10B	0.0327	0.5402	0.6642	0.197*	
H10C	0.0216	0.6246	0.5712	0.197*	
C11	0.0905 (2)	0.5998 (3)	0.9306 (4)	0.1116 (12)	
H11A	0.1338	0.6261	0.9942	0.167*	
H11B	0.1028	0.5406	0.9202	0.167*	
H11C	0.0359	0.6067	0.9770	0.167*	
C12	0.06544 (18)	0.7341 (2)	0.7915 (4)	0.1009 (10)	
H12A	0.0639	0.7611	0.6926	0.151*	
H12B	0.1073	0.7615	0.8565	0.151*	
H12C	0.0104	0.7384	0.8371	0.151*	
C13	0.24757 (12)	0.67031 (14)	0.7790 (2)	0.0550 (5)	
H13	0.2388	0.7024	0.8668	0.066*	
C14	0.32996 (11)	0.66083 (12)	0.7260 (2)	0.0463 (5)	
C15	0.40013 (12)	0.70132 (13)	0.8100 (2)	0.0512 (5)	
H15	0.3875	0.7357	0.8932	0.061*	
C16	0.54326 (13)	0.73660 (13)	0.8642 (3)	0.0547 (5)	
H16A	0.5162	0.7797	0.9258	0.066*	
H16B	0.5814	0.7650	0.7945	0.066*	
C17	0.59483 (12)	0.67922 (12)	0.9685 (2)	0.0456 (4)	
C18	0.67935 (12)	0.69802 (13)	1.0007 (2)	0.0509 (5)	
H18	0.7044	0.7446	0.9543	0.061*	
C19	0.72737 (13)	0.64835 (15)	1.1014 (2)	0.0581 (5)	
C20	0.81794 (16)	0.6718 (2)	1.1355 (4)	0.0818 (7)	
C21	0.69155 (16)	0.57852 (15)	1.1692 (3)	0.0685 (6)	
H21	0.7239	0.5445	1.2353	0.082*	
C22	0.60725 (16)	0.55961 (15)	1.1381 (3)	0.0706 (7)	
H22	0.5824	0.5129	1.1845	0.085*	
C23	0.55955 (14)	0.60916 (14)	1.0392 (2)	0.0571 (5)	
H23	0.5027	0.5955	1.0192	0.069*	
H1	0.4592 (18)	0.6288 (18)	0.616 (3)	0.088 (9)*	

### Atomic displacement parameters (Å^2^)

**Table:**

	*U*^11^	*U*^22^	*U*^33^	*U*^12^	*U*^13^	*U*^23^
F1A	0.0791 (18)	0.100 (2)	0.242 (5)	−0.0161 (14)	−0.055 (3)	−0.027 (2)
F2A	0.099 (2)	0.203 (4)	0.143 (3)	−0.021 (2)	−0.066 (2)	0.050 (3)
F3A	0.0563 (14)	0.225 (5)	0.149 (3)	−0.002 (2)	0.0203 (16)	−0.047 (3)
F1B	0.060 (5)	0.146 (9)	0.116 (7)	−0.030 (6)	−0.012 (5)	0.022 (7)
F2B	0.073 (5)	0.162 (10)	0.102 (6)	−0.002 (7)	−0.011 (5)	−0.046 (6)
F3B	0.075 (5)	0.130 (7)	0.151 (10)	0.046 (5)	−0.023 (7)	−0.009 (6)
O1	0.0422 (8)	0.0723 (10)	0.0606 (9)	−0.0033 (7)	0.0091 (7)	−0.0136 (8)
N1	0.0455 (9)	0.0569 (10)	0.0526 (9)	−0.0018 (7)	−0.0032 (7)	−0.0023 (8)
C1	0.0407 (10)	0.0457 (10)	0.0465 (10)	0.0016 (8)	0.0054 (8)	0.0020 (9)
C2	0.0491 (11)	0.0469 (11)	0.0462 (11)	−0.0004 (8)	0.0016 (9)	−0.0005 (9)
C3	0.0616 (13)	0.0663 (14)	0.0532 (12)	−0.0070 (10)	0.0051 (10)	−0.0139 (11)
C4	0.115 (2)	0.105 (2)	0.0502 (14)	−0.0252 (17)	0.0177 (14)	−0.0142 (14)
C5	0.0966 (19)	0.0724 (17)	0.0909 (19)	0.0110 (14)	0.0060 (15)	−0.0301 (15)
C6	0.0825 (18)	0.114 (2)	0.0805 (17)	−0.0190 (16)	−0.0042 (14)	−0.0440 (17)
C7	0.0446 (11)	0.0576 (12)	0.0536 (11)	−0.0041 (9)	−0.0025 (9)	−0.0021 (10)
C8	0.0420 (10)	0.0612 (13)	0.0586 (12)	0.0046 (9)	0.0044 (9)	−0.0025 (10)
C9	0.0438 (11)	0.0861 (17)	0.0744 (15)	0.0031 (11)	0.0112 (10)	−0.0033 (13)
C10	0.0439 (14)	0.200 (4)	0.151 (3)	−0.0211 (19)	0.0191 (17)	−0.066 (3)
C11	0.0784 (19)	0.144 (3)	0.114 (3)	0.0155 (19)	0.0436 (18)	0.036 (2)
C12	0.0669 (17)	0.107 (2)	0.130 (3)	0.0267 (16)	0.0250 (17)	−0.003 (2)
C13	0.0492 (11)	0.0631 (13)	0.0528 (11)	0.0048 (9)	0.0041 (9)	−0.0113 (10)
C14	0.0426 (10)	0.0490 (11)	0.0473 (10)	0.0027 (8)	0.0008 (8)	−0.0026 (9)
C15	0.0521 (12)	0.0519 (11)	0.0494 (11)	0.0026 (9)	0.0001 (9)	−0.0063 (9)
C16	0.0508 (11)	0.0525 (12)	0.0607 (12)	−0.0066 (9)	−0.0058 (9)	0.0001 (10)
C17	0.0476 (10)	0.0460 (11)	0.0433 (10)	−0.0035 (8)	0.0035 (8)	−0.0069 (8)
C18	0.0475 (11)	0.0524 (12)	0.0530 (11)	−0.0068 (9)	0.0040 (9)	−0.0036 (9)
C19	0.0515 (11)	0.0658 (14)	0.0569 (12)	0.0044 (10)	−0.0030 (9)	−0.0076 (11)
C20	0.0551 (13)	0.0978 (19)	0.0921 (18)	0.0080 (13)	−0.0109 (13)	−0.0007 (15)
C21	0.0743 (16)	0.0650 (15)	0.0655 (15)	0.0072 (12)	−0.0106 (12)	0.0095 (12)
C22	0.0817 (17)	0.0594 (14)	0.0706 (15)	−0.0142 (12)	−0.0044 (13)	0.0155 (12)
C23	0.0558 (12)	0.0585 (13)	0.0570 (12)	−0.0127 (10)	−0.0018 (10)	0.0007 (11)

### Geometric parameters (Å, º)

**Table:**

F1A—C20	1.313 (4)	C7—C8	1.390 (3)
F2A—C20	1.318 (4)	C8—C13	1.381 (3)
F3A—C20	1.290 (4)	C8—C9	1.540 (3)
F1B—C20	1.315 (6)	C9—C10	1.514 (4)
F2B—C20	1.296 (6)	C9—C12	1.521 (4)
F3B—C20	1.282 (6)	C9—C11	1.522 (4)
O1—C1	1.355 (2)	C13—C14	1.389 (3)
N1—C15	1.273 (2)	C14—C15	1.456 (3)
N1—C16	1.457 (3)	C16—C17	1.506 (3)
C1—C2	1.400 (3)	C17—C18	1.381 (3)
C1—C14	1.406 (3)	C17—C23	1.387 (3)
C2—C7	1.392 (3)	C18—C19	1.386 (3)
C2—C3	1.542 (3)	C19—C21	1.376 (3)
C3—C6	1.528 (3)	C19—C20	1.491 (3)
C3—C4	1.531 (3)	C21—C22	1.376 (3)
C3—C5	1.535 (4)	C22—C23	1.373 (3)
			
C15—N1—C16	118.71 (18)	C18—C17—C16	119.59 (17)
O1—C1—C2	119.84 (17)	C23—C17—C16	122.23 (17)
O1—C1—C14	119.60 (17)	C17—C18—C19	120.85 (19)
C2—C1—C14	120.55 (16)	C21—C19—C18	120.2 (2)
C7—C2—C1	116.51 (17)	C21—C19—C20	120.6 (2)
C7—C2—C3	121.84 (18)	C18—C19—C20	119.2 (2)
C1—C2—C3	121.64 (17)	F3B—C20—F3A	54.5 (8)
C6—C3—C4	107.3 (2)	F3B—C20—F2B	105.4 (10)
C6—C3—C5	107.4 (2)	F3A—C20—F2B	133.2 (5)
C4—C3—C5	110.5 (2)	F3B—C20—F1A	133.6 (5)
C6—C3—C2	111.79 (18)	F3A—C20—F1A	107.0 (4)
C4—C3—C2	109.91 (19)	F2B—C20—F1A	52.9 (9)
C5—C3—C2	109.89 (19)	F3B—C20—F1B	105.1 (10)
C8—C7—C2	124.77 (19)	F3A—C20—F1B	55.4 (8)
C13—C8—C7	116.71 (17)	F2B—C20—F1B	103.0 (10)
C13—C8—C9	119.89 (19)	F1A—C20—F1B	54.8 (7)
C7—C8—C9	123.37 (19)	F3B—C20—F2A	55.3 (8)
C10—C9—C12	108.2 (3)	F3A—C20—F2A	106.3 (3)
C10—C9—C11	109.6 (3)	F2B—C20—F2A	54.8 (8)
C12—C9—C11	108.5 (3)	F1A—C20—F2A	104.5 (4)
C10—C9—C8	112.0 (2)	F1B—C20—F2A	132.6 (4)
C12—C9—C8	110.2 (2)	F3B—C20—C19	113.8 (5)
C11—C9—C8	108.3 (2)	F3A—C20—C19	112.6 (3)
C8—C13—C14	121.71 (19)	F2B—C20—C19	114.2 (4)
C13—C14—C1	119.70 (18)	F1A—C20—C19	112.7 (2)
C13—C14—C15	118.93 (17)	F1B—C20—C19	114.2 (4)
C1—C14—C15	121.36 (16)	F2A—C20—C19	113.2 (3)
N1—C15—C14	123.01 (18)	C19—C21—C22	119.2 (2)
N1—C16—C17	113.12 (16)	C23—C22—C21	120.5 (2)
C18—C17—C23	118.13 (19)	C22—C23—C17	121.1 (2)
			
O1—C1—C2—C7	177.52 (18)	C16—N1—C15—C14	178.69 (18)
C14—C1—C2—C7	−1.8 (3)	C13—C14—C15—N1	175.7 (2)
O1—C1—C2—C3	−1.6 (3)	C1—C14—C15—N1	−3.9 (3)
C14—C1—C2—C3	179.11 (19)	C15—N1—C16—C17	107.5 (2)
C7—C2—C3—C6	0.3 (3)	N1—C16—C17—C18	148.23 (18)
C1—C2—C3—C6	179.3 (2)	N1—C16—C17—C23	−34.4 (3)
C7—C2—C3—C4	119.4 (2)	C23—C17—C18—C19	−0.2 (3)
C1—C2—C3—C4	−61.6 (3)	C16—C17—C18—C19	177.26 (19)
C7—C2—C3—C5	−118.9 (2)	C17—C18—C19—C21	0.9 (3)
C1—C2—C3—C5	60.2 (3)	C17—C18—C19—C20	−178.8 (2)
C1—C2—C7—C8	0.0 (3)	C21—C19—C20—F3B	42.0 (11)
C3—C2—C7—C8	179.0 (2)	C18—C19—C20—F3B	−138.3 (10)
C2—C7—C8—C13	1.8 (3)	C21—C19—C20—F3A	101.8 (4)
C2—C7—C8—C9	−176.4 (2)	C18—C19—C20—F3A	−78.5 (4)
C13—C8—C9—C10	177.7 (3)	C21—C19—C20—F2B	−79.1 (12)
C7—C8—C9—C10	−4.3 (4)	C18—C19—C20—F2B	100.6 (12)
C13—C8—C9—C12	57.2 (3)	C21—C19—C20—F1A	−137.1 (4)
C7—C8—C9—C12	−124.7 (3)	C18—C19—C20—F1A	42.6 (5)
C13—C8—C9—C11	−61.3 (3)	C21—C19—C20—F1B	162.7 (10)
C7—C8—C9—C11	116.7 (3)	C18—C19—C20—F1B	−17.6 (11)
C7—C8—C13—C14	−1.6 (3)	C21—C19—C20—F2A	−18.8 (5)
C9—C8—C13—C14	176.5 (2)	C18—C19—C20—F2A	160.9 (4)
C8—C13—C14—C1	−0.1 (3)	C18—C19—C21—C22	−1.2 (4)
C8—C13—C14—C15	−179.8 (2)	C20—C19—C21—C22	178.5 (2)
O1—C1—C14—C13	−177.42 (18)	C19—C21—C22—C23	0.8 (4)
C2—C1—C14—C13	1.9 (3)	C21—C22—C23—C17	−0.1 (4)
O1—C1—C14—C15	2.3 (3)	C18—C17—C23—C22	−0.2 (3)
C2—C1—C14—C15	−178.41 (18)	C16—C17—C23—C22	−177.6 (2)

### Hydrogen-bond geometry (Å, º)

*Cg*1 and *Cg*2 are the centroids of the C1/C2/C7/C8/C13/C14 and C17–C23 rings, respectively.

**Table:**

*D*—H···*A*	*D*—H	H···*A*	*D*···*A*	*D*—H···*A*
O1—H1···N1	0.92 (3)	1.73 (3)	2.587 (2)	154 (3)
C16—H16*B*···*Cg*2^i^	0.97	2.77	3.705 (3)	162
C21—H21···*Cg*1^ii^	0.93	2.85	3.631 (3)	143

Symmetry codes: (i) *x*, −*y*+3/2, *z*−1/2; (ii) −*x*+1, −*y*+1, −*z*+2.
